# Osseous Metaplasia in Castleman's Disease: A Case Report

**DOI:** 10.1155/2012/674870

**Published:** 2012-05-09

**Authors:** Maral Mokhtari, Perikala Vijayananda Kumar

**Affiliations:** Pathology Department, Shiraz University of Medical Sciences, Shiraz 71348-53185, Iran

## Abstract

*Background*. Castleman's disease is a benign lymphoproliferative disorder. The disease may be localized or multicentric. Three histologic variants are recognized: hyaline vascular, plasma cell, and mixed types. *Case*. A 20-year-old man presented with large left axillary mass. The histologic examination of the resected mass showed follicular pattern with large nodules of mantle cells arranged concentrically around atrophic and vascularized germinal centers. There was also some benign-appearing bone trabeculae interspersed with lymphoid tissue. The diagnosis of Castleman's disease, hyaline-vascular type with osseous metaplasia, was made.

## 1. Introduction

Castleman's disease (CD) is first described by Castleman in 1956 in a mediastinal lymph node [1, 2]. It is characterized by benign and massive proliferation of lymphoid tissue [1, 3]. Three histologic variants of CD are recognized; hyaline-vascular type (75–90%), plasma cell type (10–25%) and mixed type (1–4%) [1, 4].

The disease may be unicentric or multicentric. The hyaline-vascular type is usually unicentric and pauci symptomatic, but plasma cell type and mixed type are usually multicentric, associated with fever, anemia, weight loss, and skin rash with a more aggressive behavior [1, 2].

The exact etiology of CD is unclear, but immunosuppression, viral infection, autoimmunity, and chronic inflammation are proposed to have a role in disease development [5]. Among these, there is a strong evidence of human herpes virus-8 (HHV-8) infection as the etiologic agent of some form of CD [1].

Herein we present a case of localized hyaline-vascular type of CD presented as an axillary lymphadenopathy showing osseous metaplasia.

## 2. Case

A 20-year-old man referred to surgery department of Faghihi hospital, affiliated to Shiraz University of medical science, due to painless left axillary mass for 2 months duration. His past medical history was unremarkable. On physical examination he had stable vital signs. A firm mass was palpated at the left axillary area measuring about 8 × 7 centimeter (cm). There was no evidence of hepatosplenomegaly and generalized lymphadenopathy.

**Figure 1:**
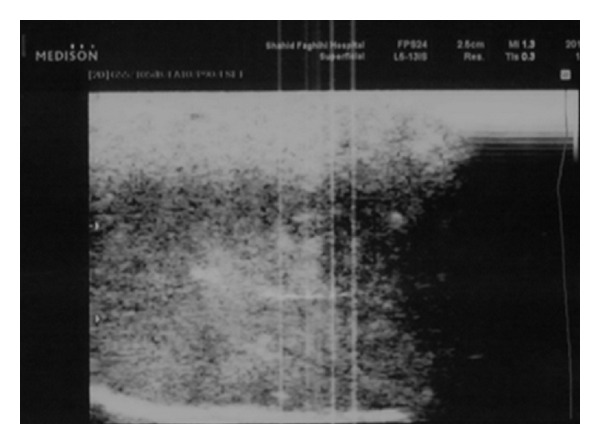
Left axillary ultrasound shows a well-defined mass with area of punctuate calcification.

Left axillary ultrasound examination showed a well-defined superficial mass with area of punctate calcification with no evidence of hemorrhage and necrosis, suggestive of an enlarged lymph node (Figure 1).

**Figure 2:**
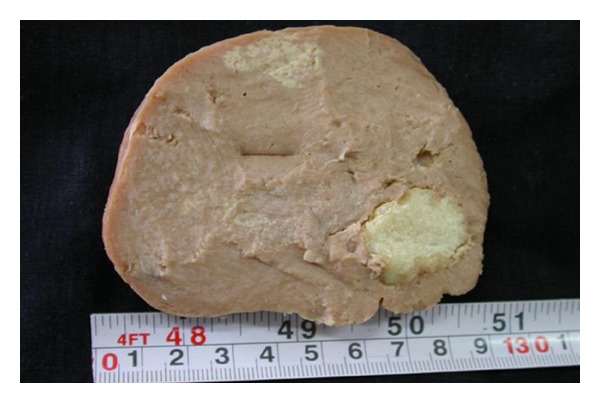
Enlarged axillary lymph node shows areas of ossification.

**Figure 3:**
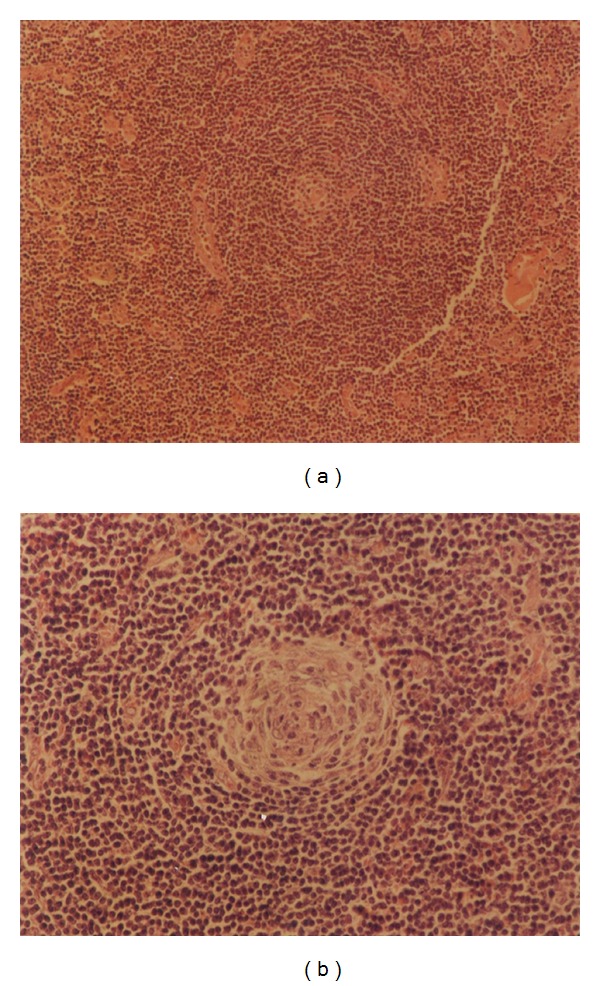
Lymph node shows large nodules of mantle cells arranged concentrically around atrophic and vascularized germinal centers, hematoxylin, and eosin, ×100 and ×400.

**Figure 4:**
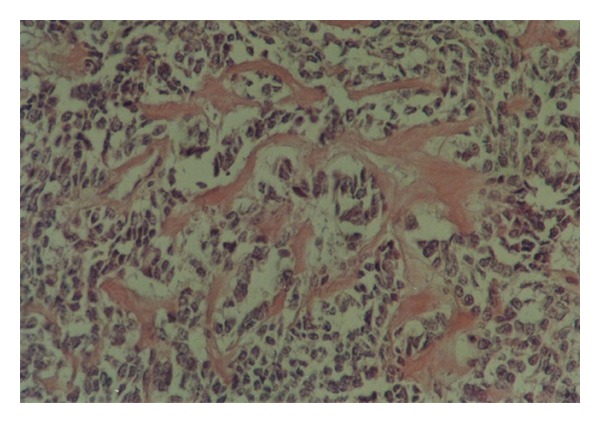
Benign-Appearing bone trabeculae interspersed with lymphoid tissue, hematoxylin, and eosin, ×400.

The patient's lab data including complete blood count with differential and biochemical tests were within normal limits. Chest X-ray and abdominal ultrasound were normal. The left axillary mass was excised. On gross inspection, there was a firm creamy brown lymph node, measuring 8 × 7 × 5 cm with focal areas of white discoloration and hard consistency (Figure 2). Histologic examination revealed follicular pattern with large nodules of mantle cells arranged concentrically around atrophic and vascularized germinal centers (Figures 3(a) and 3(b)). There was also some benign-appearing bone trabeculae interspersed with lymphoid tissue (Figure 4).

The diagnosis based on histologic examination was Castleman's disease, hyaline-vascular type with osseous metaplasia.

## 3. Discussion

CD is a benign lymphoproliferative disorder [1–6]. It is variously named as angiofollicular hyperplasia, giant lymph node hyperplasia, angiomatous lymphoid hyperplasia, localized nodal hyperplasia, and lymphoid hamartoma [3, 5]. CD has a wide age distribution ranging from adolescence to seven decades [1–3].

 In 1972 Keller et al. [7] divided CD in three histologic types: hyaline vascular type (75–90%), plasma cell type (10–25%), and mixed type (1–4%) [1, 4]. The hyaline-vascular type is characterized by small-to medium-sized follicles that contain small blood vessels that penetrate atrophic germinal centers [1, 8]. The mantle zone lymphocytes arrange in concentric layering at the periphery of follicles resulting in an onion-skin appearance [9]. The interfollicular stroma is also vascular [1]. While plasma cell type is characterized by proliferation of plasma cells in interfollicular tissue [1, 9].

According to clinical presentation CD may be unicentric or multicentric [1–6, 9, 10]. The unicentric form is usually of hyaline-vascular type, pauci symptomatic with favorable prognosis and commonly involves the mediastinum [3, 4]. Most cases of multicentric CD are of plasma cell type, associated with anemia, fever, hyperglobulinemia, hypoalbuminemia, and increased erythrocyte sedimentation rate [1–4, 9]. Another rare new histologic variant of multicentric CD is plasmablastic type which runs an aggressive course with higher incidence of progression to lymphoma or Kaposi sarcoma [5, 6].

The etiology of CD is unclear. There is a strong evidence of HHV-8 participation in development of multicentric CD [1, 6]. It is hypothesized that HHV-8 via production of Interleukin 6 (IL-6) is responsible for lymphoplasmacytic and vascular proliferation, and use of blocking antibody against IL-6 receptor may result in symptom resolution [1].

In our case osseous metaplasia was observed in a lymph node involved by hyaline-vascular type of CD. Among hematologic malignancies, amyloid-producing dyscrasia and diffuse large B-cell lymphoma were reported to produce heterotopic bones [10]. Two theories for this phenomenon are proposed: (i) tumor cells directly differentiate into osteoblasts, (ii) tumor cells secrete substances which induce osteoblastic differentiation in adjacent mesenchymal cells. One of these suggested substances that induce osseous metaplasia in epithelial tumors is bone morphogenic protein (BMP) [10]. BMPs are members of transforming growth factor *β* (TGF-*β*) which stimulates differentiation of mesenchymal cells to osteoblasts through binding to appropriate receptor [11]. Bull et al. [12] demonstrated that HHV-8 will downregulate BMP-4 (an osteogenic factor), so osseous metaplasia in CD may be produced by other substances. IL-6 is may be the responsible factor. Although this interleukin is considered as a bone resorption factor, [13, 14] it may play a role in bone formation and osteoblast proliferation, too [10, 13]. Also amyloid deposit may be seen in CD [9], and this could be site for osseous metaplasia, but no amyloid deposit was seen in our case.

The differential diagnosis of CD is non-Hodgkin's lymphoma (follicular, mantle cell, and marginal zone B cell lymphoma), Hodgkin's lymphoma [8], and a variety of reactive lymphadenopathies such as rheumatoid arthritis, Sjogren, HIV, and drug sensitivity [6]. Unicentric CD is cured by surgical resection, but the course of multicentric CD is chronic, requiring continuous therapy and it may progress to malignant lesions such as lymphoma, plasma cell dyscrasia, or Kapos' i sarcoma [6, 9].

## 4. Conclusion

To our knowledge this is the first case of CD with osseous metaplasia although the exact osteogenic substance is not clear.
